# CystiSim – An Agent-Based Model for *Taenia solium* Transmission and Control

**DOI:** 10.1371/journal.pntd.0005184

**Published:** 2016-12-16

**Authors:** Uffe Christian Braae, Brecht Devleesschauwer, Sarah Gabriël, Pierre Dorny, Niko Speybroeck, Pascal Magnussen, Paul Torgerson, Maria Vang Johansen

**Affiliations:** 1 Section for Parasitology and Aquatic Diseases, Department of Veterinary Disease Biology, Faculty of Health and Medical Sciences, University of Copenhagen, Frederiksberg, Denmark; 2 Department of Public Health and Surveillance, Scientific Institute of Public Health (WIV-ISP), Brussels, Belgium; 3 Department of Biomedical Sciences, Institute of Tropical Medicine, Antwerp, Belgium; 4 Department of Veterinary Public Health and Food Safety, Faculty of Veterinary Medicine, Ghent University, Merelbeke, Belgium; 5 Department of Virology, Parasitology and Immunology, Faculty of Veterinary Medicine, Ghent University, Merelbeke, Belgium; 6 Institute of Health and Society (IRSS), Université catholique de Louvain, Brussels, Belgium; 7 Centre for Medical Parasitology, Faculty of Health and Medical Sciences, University of Copenhagen, Copenhagen, Denmark; 8 Section of Epidemiology, Vetsuisse Faculty, University of Zurich, Zurich, Switzerland; University of Florida, UNITED STATES

## Abstract

*Taenia solium* taeniosis/cysticercosis was declared eradicable by the International Task Force for Disease Eradication in 1993, but remains a neglected zoonosis. To assist in the attempt to regionally eliminate this parasite, we developed **cystiSim**, an agent-based model for *T*. *solium* transmission and control. The model was developed in R and available as an R package (http://cran.r-project.org/package=cystiSim). **cystiSim** was adapted to an observed setting using field data from Tanzania, but adaptable to other settings if necessary. The model description adheres to the Overview, Design concepts, and Details (ODD) protocol and consists of two entities—pigs and humans. Pigs acquire cysticercosis through the environment or by direct contact with a tapeworm carrier's faeces. Humans acquire taeniosis from slaughtered pigs proportional to their infection intensity. The model allows for evaluation of three interventions measures or combinations hereof: treatment of humans, treatment of pigs, and pig vaccination, and allows for customary coverage and efficacy settings. **cystiSim** is the first agent-based transmission model for *T*. *solium* and suggests that control using a strategy consisting of an intervention only targeting the porcine host is possible, but that coverage and efficacy must be high if elimination is the ultimate goal. Good coverage of the intervention is important, but can be compensated for by including an additional intervention targeting the human host. **cystiSim** shows that the scenarios combining interventions in both hosts, mass drug administration to humans, and vaccination and treatment of pigs, have a high probability of success if coverage of 75% can be maintained over at least a four year period. In comparison with an existing mathematical model for *T*. *solium* transmission, **cystiSim** also includes parasite maturation, host immunity, and environmental contamination. Adding these biological parameters to the model resulted in new insights in the potential effect of intervention measures.

## Author Summary

*Taenia solium* is the leading cause of preventable epilepsy and the highest ranking foodborne parasite in terms of disease burden worldwide. Currently there are no large scale control programmes implemented against *T*. *solium*, but efficacious intervention tools are there, making control programmes the next step forward. Because of the zoonotic properties of the parasite, existing in both humans and pigs, a combination of intervention tools is likely to be needed. **cystiSim** is an agent-based disease model that provides insight into which intervention tools, and the frequency of administration of these tools, are needed to yield an effect on disease prevalence. **cystiSim** is a valuable tool in designing future control programmes and will assist in the elimination of *T*. *solium* as a public health problem.

## Introduction

The zoonotic tapeworm *Taenia solium* is a problem in both health and agricultural sectors in many developing countries in North and South America [1], sub-Saharan Africa [2], and Asia [3]. *Taenia solium* is transmitted between humans and pigs, but detailed knowledge about the transmission dynamics is scarce. Human tapeworm carriers (affected by taeniosis) excrete *T*. *solium* eggs in their stool, which can infect pigs (causing porcine cysticercosis) if ingested either by coprophagia or by environmental contamination through water [4] or feedstuff [5]. Dung beetles have been suggested to contribute to the dissemination of *Taenia* eggs as biological vectors. *Taenia* eggs can survive in the digestive system of beetles [6], and the presence of obligate dung beetle nematodes, has been associated with both exposure and infection of *T*. *solium* in pigs [7]. Other insects such as blowflies have been demonstrated to transmit viable *Taenia hydatigena* eggs from dog faeces to sheep or pigs [8]. Humans acquire taeniosis by consuming infected pork that is inadequately cooked. Lack of sanitation, poor hygiene, and consumption of contaminated food can cause humans to become accidental intermediate hosts (human cysticercosis) if *T*. *solium* eggs are ingested. This can lead to neurocysticercosis if the parasite larvae establish in the central nervous system.

*Taenia solium* taeniosis/cysticercosis was declared eradicable by the International Task Force for Disease Eradication in 1993, but remains a neglected zoonosis due to the limited information about its transmission, lack of sensitive diagnostic tools and treatments, and the lack of validated intervention packages [9]. Several intervention tools have been tried such as mass administration of an anthelminthic to people [10–16], treatment of pigs [17], pig vaccination [18,19], health education [20–22], and one attempt to combine treatment of pigs and humans [23]. Despite this, control has been unsuccessful and unsustainable, which now calls for an algorithm with a combination of intervention tools for optimal chance of control. Testing intervention tools in the field is time consuming and expensive. Mathematical and computational models are, although theoretical, fast and cheap to implement, and can yield indications as to which intervention tool, or combination hereof, and at which frequency, will prove most useful in obtaining control.

Kyvsgaard et al. [24] developed a compartmental transmission model for *T*. *solium*, but the model was based on data from different study sites in Latin America and lacked age structures. To our knowledge no agent-based model exists for *T*. *solium*. An agent-based model allows for flexible modelling of complex dynamics between individuals and the environment, and allows for *in silico* testing of intervention tools for control implementation. The aim of this study was to design a generic agent-based model to provide insight into the transmission dynamics of taeniosis and porcine cysticercosis, and subsequently explore the effect of feasible interventions to be used in the control of *T*. *solium* in sub-Saharan Africa.

## Methods

### CystiSim—model description

The model, **cystiSim**, was developed in the statistical programme language R (R Core Team 2016) and published as an R package [25]. The model description adheres to the ODD (Overview, Design concepts, Details) protocol for describing agent-based models [26].

#### Purpose

**cystiSim** was designed to explore the transmission aspects that perpetuate *T*. *solium* between human and porcine hosts, and the effects of potential intervention tools implementable in the control of *T*. *solium*.

#### Entities, state variables, and scale

The model consists of two entities, i.e., humans and pigs. The human agent is defined by seven state variables: sex (male/female), age in months (integer), infection with a mature *Taenia* (yes/no), infection with an immature *Taenia* (yes/no), time since infection (integer), environmental contamination (yes/no), and time since environmental contamination (integer). The pig agent is defined by eight state variables: age in months (integer), infection with mature cysticerci (yes/no), infection with immature cysticerci (yes/no), infection intensity (high/low/uninfected), time since infection (integer), residual duration of immunity (integer), time since vaccination (integer), and slaughter status (yes/no). The model is a discrete time model where each increment of time represents one month. There is no explicit modelling of space.

#### Process overview and scheduling

**Fig 1:**
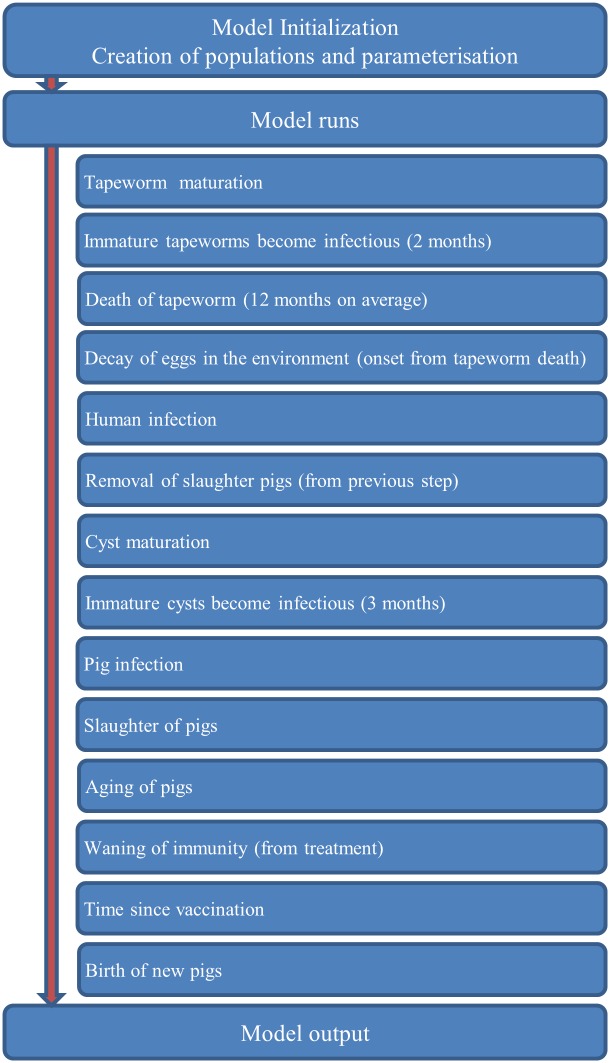
Flow chart of the process overview in cystiSim. All tapeworm carriers with a mature worm can transmit the parasite to pigs based on direct or indirect transmission (Fig 2). Direct transmission leads to high intensity infections in pigs, a simulation of coprophagia. Denoting the direct transmission probability from humans to pigs as *m2p* and the number of humans carrying a mature tapeworm as *HT*, the probability that a susceptible pig gets infected through the direct transmission route is 1 − (1 − *m2p*)^*HT*^ Indirect transmission leads to low intensity infections in pigs and constitutes the environmental contamination. Denoting the indirect transmission probability from humans to pigs as *e2p* and the number of contaminated environments as *EN*, the probability that a susceptible pig gets infected through the indirect transmission route is 1 − (1 − *e2p*)^*EN*^ Pigs do not revert from infectious to non-infectious over time, primarily based on their relative short lifespan, but pigs can go from a low intensity infection to a high intensity infection if they come into contact with a tapeworm carrier (direct transmission). Slaughtered pigs can transmit the infection based on whether they have high infection intensities or low infection intensities. Denoting the transmission probability from heavily and lightly infected pigs to humans as *ph2m* and *pl2m*, respectively, and the number of heavily and lightly infected pigs as *PIH* and *PIL*, respectively. The probability that a susceptible human gets infected through any route is 1 − [(1 − *ph2m*)^*PIH*^] * [(1 − *pl2m*)^*PIL*^] If deemed appropriate, the infection probability of susceptible humans can be made age-dependent by specifying the intercept and slope of a logistic regression model. Age-dependent susceptibility implicitly covers all factors leading to changes in infection probability within the human host such as change in immunity/resistance, eating habits, and risky behaviour. **cystiSim** allows for interventions to be tailored in terms of treatment intervals and change of efficacy and coverage for each specific intervention tool implemented. Processes that are implicitly modelled are transmission from pigs to humans influenced by natural death of pigs, natural death of cysts, cooking of pork, and meat inspection, and for transmission from humans to pigs, the use of latrines, sanitation standards, and hygiene levels.

The model is processed in the order shown in Fig 1.

**Fig 2:**
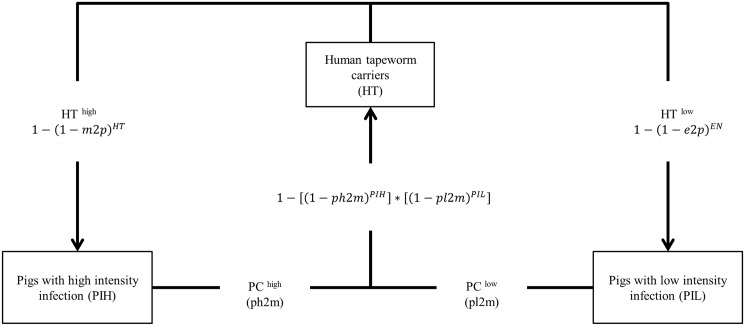
*Taenia solium* transmission pathway. Schematic overview of the different transmission pathways of *Taenia solium* incorporated in **cystiSim**. Tapeworm carriers (HT) transmit the parasite to pigs based on parameters for direct (1 − (1 − *m2p*)^*HT*^)or indirect (1 − (1 − *e2p*)^*EN*^) transmission where EN denotes the environmental contamination. Direct transmission leads to high intensity infections in pigs (PIH), a simulation of coprophagia. Indirect transmission leads to low intensity infections in pigs (PIL) and constitutes the environmental contamination. Pigs transmit the infection based on two parameters, for high infection intensities (ph2m) or low infection intensities (pl2m).

#### Design concepts: Basic principles

The model is driven by the interaction between pigs and humans. The structure and size of the human population was created based on the 2012 census data for 0–79 year olds from the districts Mbeya and Mbozi in Tanzania [27]. The human population stays constant throughout the model without aging or mortality occurring. The model was constructed in this way since we do not expect the model to simulate a period long enough for the composition of the human population to change in reality. However, this does allow for the possibility to have age dependent infection probabilities. We furthermore assumed *Taenia* spp. not to induce immunity in human hosts after tapeworm clearance.

An infected person can either infect pigs directly (coprophagia by pigs) or indirectly (through environmental contamination of eggs). Once a person is infected, the tapeworm will remain immature for two months, and during this stage it is non-infectious to pigs. After this period the tapeworm becomes infectious to pigs and begins releasing eggs into the environment. Each tapeworm is assumed to live 12 months and release approximately 1,500,000 eggs into the environment. The actual lifespan of a *T*. *solium* tapeworm is unknown, but studies have indicated that the tapeworm is not long lived as anecdotally mentioned in the literature [1]. An adult tapeworm can approximately excrete 50,000 eggs per day [28], which is equivalent to 1,500,000 eggs per month. A person can only harbour one tapeworm at the time in **cystiSim** [29]. The environmental contamination of eggs was modelled as an attribute of each individual person with taeniosis and is not a spatial characteristic. The environment will stay infective as long as the human has an adult tapeworm. Once the tapeworm dies, the environmental contamination will start to decrease based on a probability of egg decay. Contact between pigs and tapeworm carriers/environment occur at random.

#### Design concepts: Emergence and observation

The main emergent outputs of **cystiSim** are the prevalence of taeniosis, the prevalence of porcine cysticercosis, and the proportion heavy versus light infected pigs. These outputs are observed at each time point and used to assess the validity of the model parameters in the baseline (i.e., no intervention) scenario, and to assess the effects of the interventions.

#### Design concepts: Interaction and stochasticity

Direct interactions between pigs and humans (including their environment) dominate the *T*. *solium* transmission process. Several of the main processes are assumed to be stochastic, i.e., the infection of new human hosts, the infection of new porcine hosts, the decay of eggs, and the slaughter of pigs. In the intervention models, the selection of targeted individuals and the effectiveness of treatment are also stochastic variables. All stochastic processes are modelled as binomial distributions.

#### Other design concepts

The individuals do not have adaptive traits, i.e., they do not change their behaviour in response to changes in themselves or their environment. As a result, other design concepts such as objectives, learning, prediction, and sensing are not present. Collectives are also not included.

#### Sub-models

In our model, the decay of eggs in the environment is time based with an exponential decay function with a rate parameter of 0.268, derived from the assumption that 80% of *Taenia* eggs exposed in the environment are non-infective after six months [30]. We assumed that all eggs are non-infective after nine months (truncated at nine months).

Infected pigs will start in the immature cyst stage, which lasts three months [31]. During this period pigs are infected, but not infectious to humans. After this period the infected pigs move to the mature stage and are now infectious to humans when slaughtered.

### Intervention tools

#### Mass drug administration to humans

The **cystiSim** package contains a generic function for mimicking anthelmintic treatment of humans. The user is able to define the level of coverage, the treatment efficacy, and the age span within which the treatment is applied (e.g., only school-aged children [SAC]). Based on these settings, effective treatment is randomised over the eligible individuals, resulting in the loss of the (immature or mature) tapeworm, and the initiation of the decay of environmental contamination.

#### Anthelmintic treatment of pigs

The **cystiSim** package contains a generic function for mimicking mass drug administration of an anthelminthic to pigs. As for human treatment, the user is able to define the level of coverage, the treatment efficacy, and the age span of eligible pigs. Treatment can first be given to pigs at the age of two months, which is therefore the default minimum age. Based on these settings, effective treatment is randomized over the eligible pigs, resulting in the loss of the (immature or mature) cysts. The anthelminthic properties in **cystiSim** are based on the properties of oxfendazole. Pigs with porcine cysticercosis that receive treatment will therefore gain immunity after treatment, whereas there is no effect of treatment in uninfected pigs [17,32,33]. The duration of this immunity is by default set to three months, but can be changed by the user. Oxfendazole is under registration process in several sub-Saharan African countries and will soon be available as an intervention tool for porcine cysticercosis.

#### Pig vaccination

In the generic function for mimicking pig vaccination, the user is able to define the level of coverage, the vaccination efficacy, and the age span of eligible pigs. The vaccine can first be given to pigs at two months of age, which is therefore the default minimum age. The vaccine is only effective with two consecutive inoculations and is predicted to work well at maximum four months apart corresponding to the properties of the TSOL18 vaccine [34]. The TSOL18 is capable of providing almost perfect protection against porcine cysticercosis [35], and is currently on-going registration processes for use in pigs in several countries. The user is able to define the interval required between two consecutive effective inoculations, with a default of four months. The vaccine is by default assumed to provide life-long immunity, but does not kill cysts present in already infected pigs [36]. If coverage is less than 100% then pigs are allocated for vaccination at random, irrespective of which pigs had been vaccinated at an earlier stage.

Because pig vaccination is recommended to be combined with an anthelmintic treatment, a generic function is available for mimicking combined anthelmintic treatment and vaccination. This function implements the same assumptions for pig anthelmintic treatment and vaccination as outlined before, but additionally ensures perfect correlation between coverage for pig anthelmintic treatment and vaccination, i.e., mimicking the fact that when pigs are caught, they receive both anthelmintic treatment and vaccination.

### Implementation of a sub-Saharan setting

#### Baseline model

The size of the porcine population was defined from the 2007/2008 agricultural census from Mbeya and Mbozi district [37] and the population structure was constructed based on the baseline data from Braae et al. [38]. This was possible because of the sampling technique used in the survey (all pigs above 2 months of age sampled). Because pigs 0–2 months of age were not sampled they were missing from the dataset. The number of piglets (0–2 months) was based on the calculated mortality rate from the rest of the population and the corresponding number of piglets was added for the months 0, 1, and 2.

To model slaughter of pigs we assumed no pigs were slaughtered during the first six months of their lives and that pigs on average were slaughtered when they were about one year old and always before reaching the age of 36 months. To mimic this, the probability of slaughter was empirically modelled based on the cumulative distribution function of a negative binomial distribution with a mean of 80 and a dispersion parameter of 0.70. Based on this the porcine population structure was constructed mimicking the age structure seen in the data. This roughly corresponded to the dataset where 7% of the pigs after the age of 6 months reached the age of 24 months. To keep the porcine population constant, the number of slaughtered pigs at each time step (month) was automatically replaced with piglets (0 months). It was assumed that pigs in reality rarely reach an age above 36 months and pigs older than that therefore have a negligible role in transmission.

#### Initialisation

Initial infection for humans and pigs were set according to district baseline data from Braae et al. [15] and Braae et al. [38], respectively, and initial infection intensity proportions in pigs were based on data from the same area by comparing lingual examination and Ag-ELISA results [4]. Positives for lingual examination were considered high intensity infections, and Ag-ELISA positives considered low intensity infection if not concurrently positive by lingual examination. The values of four transmission parameters are unknown, but are likely to differ across geographical regions. To estimate these parameters, we simulated random sets of parameters and used these to run a model for 500 months. For each "random" model, we computed the sum of squared differences between the modelled and observed taeniosis prevalence of 3.0% [15] and a porcine cysticercosis prevalence of 13% [38] on district level, and the infection intensity [4]. We retained 10,000 sets with a sum of squared differences smaller than 0.10, and used the parameter set that resulted in the lowest deviance. For a summary of the different model parameters please see S1 Table. Random subsamples were drawn from the human population dataset using a binomial distribution to reach the targeted population level and initial infection at baseline based on an age dependent coefficient derived from logistic regression of the baseline dataset on a district level. Porcine cysticercosis infected pigs were assigned at the initial stage of the model randomly, since age could not be associated with infection from the baseline dataset [38]. All pigs leaving the population after the age of six months are assumed to be slaughtered and consumed. Mortality due to other events after the age of six months was therefore ignored.

#### Intervention and elimination scenarios

In our scenarios the efficacy of the pig anthelmintic was set to 90%, based on efficacy studies of oxfendazole. Oxfendazole given orally at 30mg/kg is currently the most efficacious anthelminthic developed for treating porcine cysticercosis [39]. The drug has shown to reduce viable cysts with 50% one week after treatment [40], and render all cysts located in the muscles non-viable 12 weeks after treatment, but with less than 100% efficacy against cysts located within the brain [32]. Drug coverage varies in the different scenarios as stated. Ten intervention strategies of four years in length were simulated in **cystiSim** to investigate impact of different intervention tool options. Five long-term intervention strategies were simulated to explore when the model would predict parasite elimination (Table 1).

**Table 1:** Overview of the different scenarios simulated with cystiSim using the interventions: mass drug administration (MDA) to humans, anthelmintic treatment of pigs (ANT), and vaccination of pigs (VAC). MDA is given to school-aged children (SAC) or the entire community (All). The interval between interventions is denoted by q.

ID	Description	Age group	Coverage	Efficacy
	Four year intervention strategies			
INT-1	MDA 4q12	All	75%	90%
INT-2	MDA 4q12	SAC	90%	90%
INT-3	ANT 11q4	All	90%	90%
INT-4	ANT 11q4	All	75%	90%
INT-5	ANT + VAC 11q4	All	90%	90%
INT-6	ANT + VAC 11q4	All	75%	90%
INT-7	MDA 4q12 + ANT 11q4	SAC	90% (Humans), 75% (Pigs)	90%
INT-8	MDA 4q12 + ANT 11q4	All	75%	90%
INT-9	MDA 4q12 & ANT + VAC 11q4	SAC	90% (Humans), 75% (Pigs)	90%
INT-10	MDA 4q12 & OFZ + VAC 11q4	All	75%	90%
	Elimination strategies			
ELIM-1	MDA q12	all	90%	90%
ELIM-2	ANT q4	all	75%	90%
ELIM-3	ANT + VAC q4	all	75%	90%
ELIM-4	MDA q12 & ANT + VAC q4	SAC	90% (Humans), 75% (Pigs)	90%
ELIM-5	MDA q12 & ANT + VAC q4	all	75%	90%

#### Implementation

To capture the stochastic effects in the model, each scenario was run a 1,000 times, and the mean and 95% uncertainty interval (UI) of the human taeniosis prevalence, the porcine cysticercosis prevalence, and the pig immunity prevalence, was plotted. The control interventions were implemented after an initial burn-in phase of 200 iterations; after the end of the control interventions, the model was run for another 120 iterations (i.e., a “burn-out” phase of 10 years). We recorded the number of model runs where elimination was achieved (defined as a human taeniosis and porcine cysticercosis prevalence of zero at the end of the burn-out phase). The elimination interventions were also implemented after an initial burn-in phase of 200 iterations, but now the total intervention time was defined such that elimination was obtained in all 1000 model runs. For all runs, we recorded the time between the start of the elimination intervention and the achievement of elimination, and reported the mean and range. We used **cystiSim** version 0.1.0 [25] in R version 3.3.0 (R Core Team 2016) to run the scenarios.

## Results

### Validation of the model

To validate the parameter settings and check that **cystiSim** yielded a stabile output similar to the baseline dataset a simulation running for 2000 months, but without any interventions was performed for both Mbeya and Mbozi district and yielded no discrepancies compared to the initial datasets. Output graphs for both simulations are included in S1 Appendix, and show a good fit with the baseline data reported by Braae et al. [38]. Only scenarios for one district (Mbeya) are reported from here on, but all simulations were performed for both districts and all results for Mbozi district are available in S2 Appendix for comparison. **cystiSim** yields elimination probabilities in both pigs and humans, but only the lowest value is given henceforth.

### Scenarios with simulation of four year intervention strategies

#### Treatment of humans

**Fig 3:**
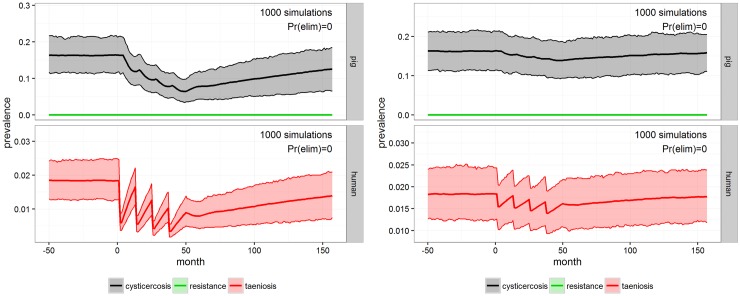
INT-1 and INT-2. Outcome of the MDA to the whole population with 75% coverage (INT-1, left) and MDA to school-aged children with 90% coverage (INT-2, right) for Mbeya district after 1000 simulations in **cystiSim**. Efficacy was set at 90% in all of the simulations. The coloured area demarcates the 95% uncertainty intervals for prevalence. The green line illustrates pig resistance towards new infections and Pr(elim) states the predicted probability of elimination occurring in the given scenario.

INT-1 targeting the whole population with four rounds of MDA resulted in relative large decreases in levels of taeniosis and decreased porcine cysticercosis prevalence during the intervention period, but with a predicted elimination probability of 0.00%. The model suggested that taeniosis prevalence quickly rebounds after MDA administration and the prevalence of both porcine cysticercosis and taeniosis relatively quickly would return to pre-intervention levels once intervention was terminated (Fig 3). INT-2 with the strategy of administrating four rounds MDA to school-aged children has according to the model little impact over the four year period both on the prevalence of taeniosis in the general population and on the prevalence of porcine cysticercosis compared to INT-1 (Fig 3). Both prevalence of taeniosis and porcine cysticercosis relatively quickly rise towards pre-intervention levels.

#### Anthelmintic treatment of pigs

**Fig 4:**
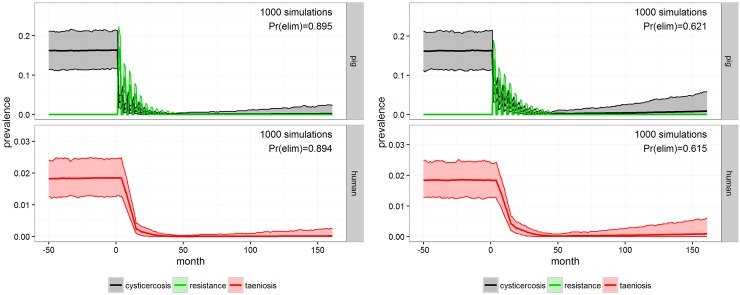
INT-3 and INT-4. Outcome of the porcine population treatment with 90% coverage (INT-3, left) and 75% coverage (INT-4, right) for Mbeya district after 1000 simulations in **cystiSim**. Efficacy was set at 90% in all of the simulations. The coloured area demarcates the 95% uncertainty intervals for prevalence. The green line illustrates pig resistance towards new infections and Pr(elim) states the predicted probability of elimination occurring in the given scenario.

INT-3 with 11 rounds of pig anthelmintic treatment shows promising results, despite the lack of other interventions e.g. a vaccine, with the prevalence of both taeniosis and porcine cysticercosis rapidly brought down (Fig 4, left), and with a predicted elimination probability of 0.89. INT-4 used the same approach as INT-3, but with a 15 percentage point lower coverage (Fig 4, right). This also resulted in rapid decreases in prevalence of both taeniosis and porcine cysticercosis, but yielded a lower probability of elimination (0.62) compared to INT-3.

#### Vaccination of pigs

**Fig 5:**
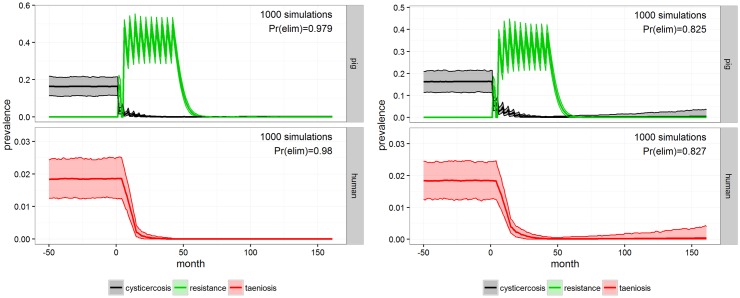
INT-5 and INT-6. Outcome of anthelmintic treatment and vaccination of the porcine population with 90% coverage (INT-5, left) and 75% coverage (INT-6, right) for Mbeya district after 1000 simulations in **cystiSim**. Efficacy was set at 90% in all of the simulations. The coloured area demarcates the 95% uncertainty intervals for prevalence. The green line illustrates pig resistance towards new infections and Pr(elim) states the predicted probability of elimination occurring in the given scenario.

The INT-5 strategy with 11 rounds of vaccination and anthelmintic treatment of pigs was highly effective in the simulation and almost managed to eliminate both diseases with a probability of 0.98 (Fig 5, left). INT-6 using the same approach as INT-5, but with a lower coverage (75%), was also highly effective (Fig 5, right), but with a lower probability of elimination (0.83). The reduction of 15 percentage points in coverage also resulted in reductions of prevalence for both taeniosis and porcine cysticercosis, but a 15% percentage point reduction in elimination probability.

#### Strategies combining pig and human interventions

**Fig 6:**
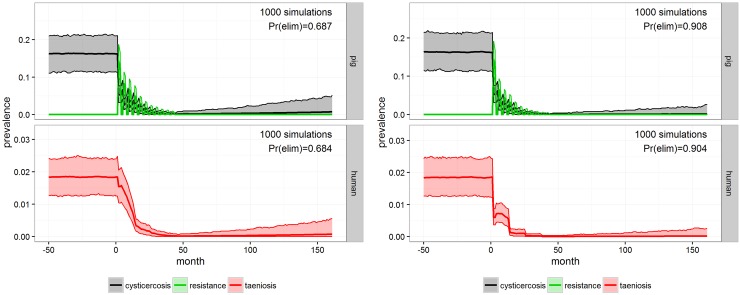
INT-7 and INT-8. Outcome of MDA to school-aged children with 90% coverage in combination with anthelmintic treatment of the porcine population with 75% coverage (INT-7, left) and outcome of INT-8 which consisted of MDA to the whole human population in combination with anthelmintic treatment of the porcine population both with 75% coverage (on the right), for Mbeya district after 1000 simulations in **cystiSim**. Efficacy was set at 90% in all of the simulations. The coloured area demarcates the 95% uncertainty intervals for prevalence. The green line illustrates pig resistance towards new infections and Pr(elim) states the predicted probability of elimination occurring in the given scenario.

When combining four rounds of MDA to school-aged children with 11 rounds of pig anthelmintic treatment and vaccination (INT-7), **cystiSim** predicted a 0.68 probability of elimination (Fig 6, left). However, if all individuals were targeted, albeit at a lower coverage (75%), but using the same modality as in INT-7 with anthelmintic treatment of pigs only, the probability of elimination was estimated to 0.90 in scenario INT-8 (Fig 6, right). Compared with just providing anthelmintic treatment to pigs at 75% coverage which yielded a probability of elimination of 0.62 (Fig 4, right), school-based MDA only provided 0.06 higher probability of elimination.

**Fig 7:**
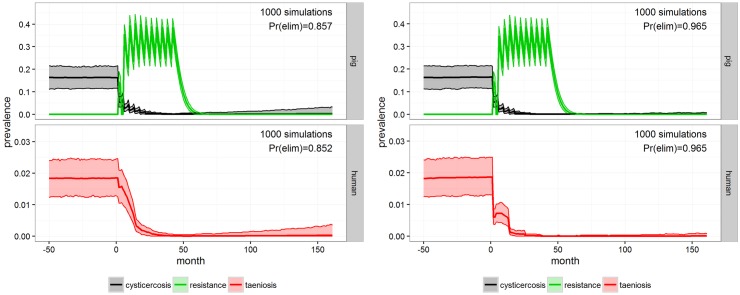
INT-9 and INT-10. Outcome of MDA to school-aged children with 90% coverage in combination with anthelmintic treatment and vaccination of the porcine population with 75% coverage (INT-9, left) and outcome of INT-10 which consisted of MDA to the whole human population in combination with anthelmintic treatment and vaccination of the porcine population both with 75% coverage (on the right), for Mbeya district after 1000 simulations in **cystiSim**. Efficacy was set at 90% in all of the simulations. The coloured area demarcates the 95% uncertainty intervals for prevalence. The green line illustrates pig resistance towards new infections and Pr(elim) states the predicted probability of elimination occurring in the given scenario.

Combining school-based MDA with coverage of 90% with anthelmintic treatment and vaccination of pigs with coverage of 75% (INT-9), **cystiSim** estimated the probability of elimination to be 0.85 (Fig 7, left). Changing the MDA to include all individuals (INT-10), but with a coverage of 75% and still combined with pig intervention as in INT-9, **cystiSim** predicted a probability of elimination of 0.97 within the four your period (Fig 7, right).

### Scenarios with simulation of elimination strategies

All elimination scenarios were performed with a set efficacy of 90% except for vaccination which was set at 95%, but since it needs to be administrated twice to be effective this also yields an overall efficacy of 90% in terms of getting successful immunisation after vaccination (Table 2). ELIM-1 showed that MDA will not be effective in terms of elimination, even if the programme is continued for 20 years, however, cystiSim did predict elimination in Mbeya district after approximately 26 (range: 10–90) years. Treatment of pigs with an anthelmintic drug every four months simulated in ELIM-2 resulted in elimination of the parasite after 49 months (range: 24–119). Adding the vaccine to the anthelmintic treatment of pigs was simulated in ELIM-3 and reduced the time to elimination with seven months (42 [20–100]) and shortened the range, compared to ELIM-2. Adding treatment of school-aged children to anthelmintic treatment and vaccination of pigs in ELIM-4 only reduced the time to elimination with two months (40 [19–100]) compared to ELIM-3. The shortest intervention period was seen in ELIM-5 where the whole human population was treated annually combined with anthelmintic treatment and vaccination of pigs every four months, which resulted in a mean of 32 (13–72) months to elimination.

**Table 2:** Duration of months until elimination of *Taenia solium* in human and porcine hosts was achieved in Mbeya district by simulation in cystiSim. Efficacy of was fixed at 90%. Coverage of mass drug administration (MDA) to school-age children (SAC) and MDA to the whole human population (All) was set to 90%. Coverage of pig interventions, vaccination (VAC) and anthelmintic treatment (ANT), was set to 75%.

Strategy	Human target group	Porcine cysticercosis Months until elimination			Taeniosis Months until elimination		
		Mean	Min	Max	Mean	Min	Max
ELIM-1 (MDA)	All	313	119	1080	310	120	1080
ELIM-2 (ANT)	Not applicable	49	24	120	51	27	124
ELIM-3 (ANT+VAC)	Not applicable	39	20	88	43	24	90
ELIM-4 (MDA & ANT+VAC)	SAC	38	20	94	41	23	107
ELIM-5 (MDA & Pig MDA+VAC)	All	30	14	84	33	17	79

## Discussion

**cystiSim**, the first agent-based transmission model for *T*. *solium*, predicts that control of *T*. *solium* using a strategy consisting of an intervention targeting the porcine host, is possible, albeit coverage and efficacy of the intervention has to be high if elimination is the ultimate goal. Good coverage of the intervention is crucial, but lower coverage can be compensated for by including an intervention targeting the human host. **cystiSim** shows that the scenarios combining interventions in both hosts, MDA to humans, and vaccination and anthelmintic treatment of pigs, have high probabilities of success if a coverage of 75% can be maintained over a four year period. So far no intervention programmes or studies have tried this modality in Africa and therefore comparison to African field data is impossible. Only one study from Africa, where Braae et al. [15] measured the effect of MDA of praziquantel given to Tanzanian school-aged children for schistosomiasis treatment in combination with 'track-and-treat' of taeniosis cases diagnosed during the study, have shown an effect on taeniosis prevalence. Garcia et al. [23] conducted a short-term intervention study in Peru targeting both hosts and measured the outcome based on EITB on pigs only. A decrease in prevalence and incidence of porcine cysticercosis was observed, but the effect of the intervention on prevalence of taeniosis was uncertain. In Laos, Okello et al. [41] showed a significant drop in taeniosis prevalence following two annual albendazole MDA campaigns, and one pig vaccination and treatment campaign. No information on the effect on porcine cysticercosis was provided.

**cystiSim** is a novel approach to try and fill the gap between the lack of knowledge about the parasites transmission dynamics and the effect of available intervention tools. In comparison with an existing mathematical model for *T*. *solium* transmission [24], **cystiSim** includes parasite maturation, host immunity, and environmental contamination. Adding these key biological parameters to the model resulted in new insights in the potential effect of intervention measures such as that the combination of vaccination and anthelmintic treatment of pigs could yield promising results as supported by Johansen et al. [42]. However, anthelmintic treatment of pigs as a standalone tool might also provide a significant effect on the reduction of *T*. *solium*, but is likely to be more effective long-term when combined with a vaccine. In simulations with high coverage percentages (90%) there was little difference between the vaccination and anthelmintic treatment of pigs, and the anthelmintic treatment of pigs only strategy. However, as coverage decreases **cystiSim** predicts the vaccination and pig anthelmintic treatment strategy to be more robust compared to the pig anthelmintic treatment only strategy. Field efficacy studies exist for both pig vaccination [36] and pig anthelmintic treatment [17], but field data investigating the effectiveness of these strategies are lacking.

In terms of eliminating *T*. *solium* from a given area, **cystiSim** predicts that the two host target strategy is the most optimal option, as the single host strategies will have to be continued for a longer period. **cystiSim** is capable of predicting elimination because the system is closed, and of a certain size—reflecting an endemic district in Tanzania. The probability of elimination is linked to the population size and the efficacy and coverage of the interventions simulated. Because **cystiSim** currently lacks a spatial structure, then, the larger the population, the more unlikely elimination will be if coverage and efficacy is not 100%, as the probability that at least one infected host remains infected will be larger. However, this does not affect the relative effect when comparing different interventions simulated in **cystiSim**, only the probability of elimination outcome. Therefore, if the population size is changed in the model, then comparing predicted probabilities of elimination with previous scenarios should be done with caution.

MDA to the whole human population might be feasible, but will be costly and will require substantial resources to keep coverage at 75%, especially when running the programme for an extended period of time. The 75% coverage used in **cystiSim** is probably quite optimistic and a drop in adherence should be expected over time, unless great effort is put into preserving high adherence levels. The 90% coverage of MDA to school-children might be more realistic as pupils are easier to locate when in school and keeping adherence at an elevated level over a longer period of time compared to adults might be less challenging [43]. However, similar results as school-based MDA in combination with anthelmintic treatment of pigs were seen in the anthelmintic treatment of pigs only strategy, questioning the relevance of implementing MDA to schoolchildren if an anthelmintic drug to pigs is available. **cystiSim** predicts that MDA on it is own is inadequate in terms of elimination *T*. *solium*, but there is an effect of the MDA when carried out over an extended period, which might make it a valid tool for control, but this of course would depend on the cost-effectiveness of such an intervention.

Simulations of four year control programmes using the Reed-Frost transmission model [24] have recently been published [42]. When comparing these results to the four year scenarios run in **cystiSim**, the scenarios in **cystiSim** are more likely to succeed although more intensive, but with a more realistic approach to coverage and efficacy. Another obvious difference is the speed at which taeniosis and porcine cysticercosis returns to pre-intervention levels. **cystiSim** predicts a slower increase in prevalence compared to the model by Kyvsgaard et al. [24] after termination of the intervention programme, and if correct, could make the impact of a four year control programme in sub-Saharan Africa greater than expected, should it be discontinued. The two models predict similar outcomes when interventions are implemented as single interventions. However, the model by Kyvsgaard et al. [24] requires the user to input degree of transmission reduction, which is difficult to estimate. Especially in terms in MDA as a single intervention approach, both models predict a rapid increase in taeniosis prevalence shortly after treatment, questioning the effect of MDA if implemented as a stand-alone tool over shorter time periods. Both models predict that although control might be possible, elimination is difficult. Several agent-based models have been developed for investigating the burden of foodborne diseases [44] and the transmission dynamics of other parasites or zoonotic diseases [45–47]. However, simply adapting existing agent-based models to fit a parasite with a complex life cycle, such as *T*. *solium*, is not straightforward. As agent-based models are designed to fit a specific purpose an adaptation of an existing model should be done with caution. The development of a new model is often better suited for the purpose.

The processes implicitly modelled in **cystiSim** such as natural death of cysts, cooking of pork, meat inspection, and use of latrines, were implicitly modelled due to the lack of data quantifying the effect of interventions involving these processes. Furthermore, potential variations in infectivity levels of humans, pigs, and contaminated environments were ignored. In time as more data become available, these processes could be explicitly incorporated into **cystiSim**.

**cystiSim** can be a valuable tool for assessing intervention strategies. However, it is important to underline that the transmission settings and parameters affecting transmission might vary substantially from region to region. In terms of predicting elimination **cystiSim** has the limitation of not taking the influx of potential carriers in the system into account. Within a small population the impact of importing just one person with taeniosis, could affect the probability of elimination substantially. The possible occurrence of large scale mortality due to African swine fever and sales of pigs leading to increased slaughter rates is not taken into account either. **cystiSim** is limited by the lack of a spatial dimension and the assumption of homogeneous mixing. Therefore clustering of the parasite is ignored although studies have shown clustering to occur [48–50]. The next logical step is to further develop **cystiSim** to include spatial distribution and pig management characteristics. Also, when studies on the effect of health education and meat inspection are available, they should be incorporated into **cystiSim**. Data on the ratio between taeniosis/porcine cysticercosis and human cysticercosis are missing, but could also be incorporated into the model making **cystiSim** capable of predicting estimations on disease burden of neurocysticercosis.

**cystiSim** was created to provide insight into the transmission dynamics of *T*. *solium* and to explore impact of potential intervention strategies and combinations hereof using data from sub-Saharan Africa. Also, **cystiSim** was designed to allow the users to customise their desired intervention strategy. We believe that this flexibility in the design will make **cystiSim** a useful tool to anybody who works with control and prevention of *T*. *solium* in endemic countries. **cystiSim** predicts that elimination is possible, but focus should now be moved towards obtaining control within a given area before elimination can be a realistic goal.

## Supporting Information

S1 Table cystiSim model parameters. (PDF)

S1 Appendix Mbeya and Mbozi baseline model. (PDF)

S2 Appendix Mbozi intervention and elimination models. (PDF)
