# Supplementary material for: CystiSim – An Agent-Based Model for Taenia solium Transmission and Control
Source: PLoS Negl Trop Dis. 2016 Dec 16;10(12):e0005184. doi: 10.1371/journal.pntd.0005184 (PMC5161321; doi:10.1371/journal.pntd.0005184)
Supplement: S1 Table — (PDF) [file pntd.0005184.s001.pdf]

S1 Table. cystiSim model parameters.

Table A. General model parameters.

Parameter	Value	Source
Adult *Taenia solium* lifespan	12 months	(Garcia et al., 2003)
Maximum number of adult *Taenia solium* harboured per person	1	(Pawlowski, 2002)
Minimum age of pork consumption	24 months	NA
Duration of immature stage for cysts	3 months	(Verástegui et al., 2000)
Duration of immature stage for adult tapeworms	2 months	(Pawlowski, 2002)
Environmental decay rate of *Taenia solium* eggs	0.268	(Ilsoe et al., 1990)
Maximum duration of environmental infectivity of *Taenia solium* eggs	9 months	(Flisser, 1994)

Table B. User-definable model parameters.

Parameter	Default value
Transmission model	
m2p (man to pig transmission probability)	NULL
e2p (environment to pig transmission probability)	NULL
ph2m (heavy infected pig to man transmission probability)	NULL
pl2m (lightly infected pig to man transmission probability)	NULL
Logistic regression intercept and slope for age-dependent susceptibility to human taeniosis	(0, 0)
Slaughter function	slaughter_nbinom
Slaughter model – negative binomial (slaughter_nbinom)	
Minimum age at slaughter	NULL
Maximum age at slaughter	NULL
Mean of probability distribution	NULL
Dispersion parameter of probability distribution	NULL
Slaughter model – binomial (slaughter_binom)	

Minimum age at slaughter	NULL
Maximum age at slaughter	NULL
Slaughter probability	NULL
Mass drug administration – humans	
Coverage	NULL
Efficacy	NULL
Minimum eligible age	0 months
Maximum eligible age	Inf
Mass drug administration – pigs	
Coverage	NULL
Efficacy	NULL
Duration of immunity following effective treatment of positive pig	3 months
Minimum eligible age	1 month
Maximum eligible age	Inf
Vaccination – pigs	
Coverage	NULL
Efficacy	NULL
Duration of immunity following successful vaccination	Inf
Maximum interval between two effective vaccinations for conveying immunity	4 months
Minimum eligible age	1 month
Maximum eligible age	Inf
Mass drug administration & vaccination – pigs	
Coverage	NULL
Efficacy of mass drug administration	NULL
Efficacy of vaccination	NULL
Duration of immunity following effective treatment of positive pig	3 months
Duration of immunity following successful vaccination	Inf
Maximum interval between two effective vaccinations for conveying immunity	4 months
Minimum eligible age	1 month
Maximum eligible age	Inf

References

Flisser, A., 1994. Taeniasis and cysticercosis due to *Taenia solium*. Prog Clin Parasitol 4, 77.

Garcia, H.H., Gonzalez, A.E., Evans, C.A.W., Gilman, R.H., Cysticercosis Working Grp, P., 2003. *Taenia solium* cysticercosis. Lancet 362, 547-556.

Ilsoe, B., Kyvsgaard, N.C., Nansen, P., Henriksen, S.A., 1990. A study on the survival of *Taenia saginata* eggs on soil in Denmark. Acta Vet. Scand. 31, 153-158.

Pawlowski, Z.S., 2002. *Taenia solium* Cysticercosis. From Basic to Clinical Sciences. CABI Publishing, Wallingford, United Kingdom, 1-14 pp.

Verástegui, M., González, A., Gilman, R.H., Gavidia, C., Falcón, N., Bernal, T., Garcia, H.H., 2000. Experimental infection model for *Taenia solium* cysticercosis in swine. Vet. Parasitol. 94, 33-44.
