# Supplementary material for: CystiSim – An Agent-Based Model for Taenia solium Transmission and Control
Source: PLoS Negl Trop Dis. 2016 Dec 16;10(12):e0005184. doi: 10.1371/journal.pntd.0005184 (PMC5161321; doi:10.1371/journal.pntd.0005184)
Supplement: S1 Appendix — (PDF) [file pntd.0005184.s002.pdf]

cystiSim—an agent-based model for *Taenia solium* transmission and control

S1 Appendix. Mbeya and Mbozi baseline model.

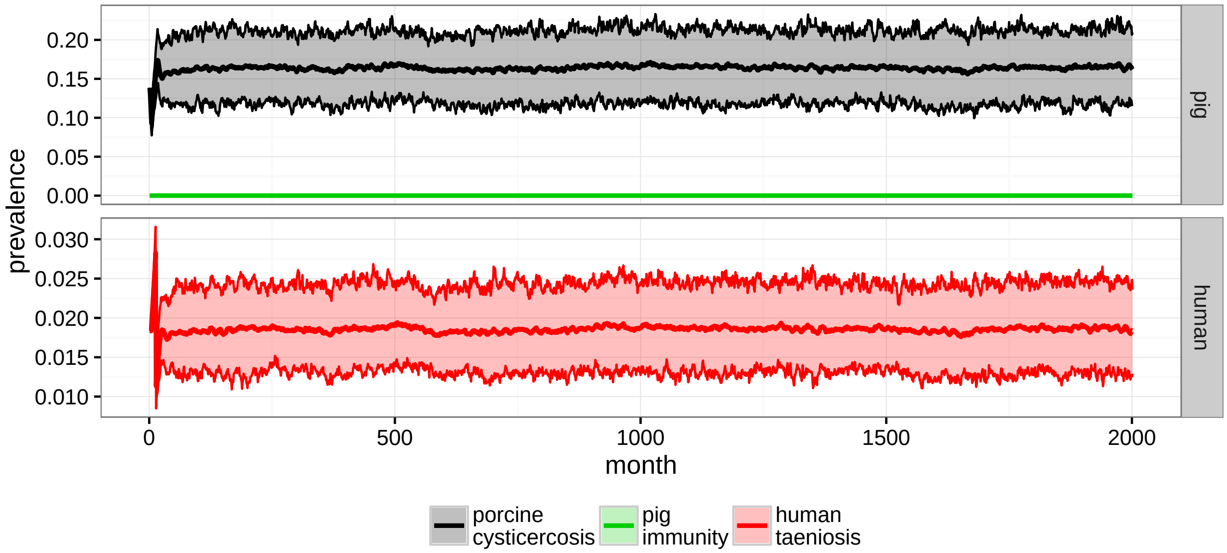

Figure 1: Mbeya baseline model; mean prevalence and 95% uncertainty interval based on 100 simulations of 2000 months each. Parameter settings: $ph2m = 0.00017$; $pl2m = 0.00015$; $m2p = 0.00007$; $e2p = 0.00023$; $age.coef = (-5, 0.03)$.

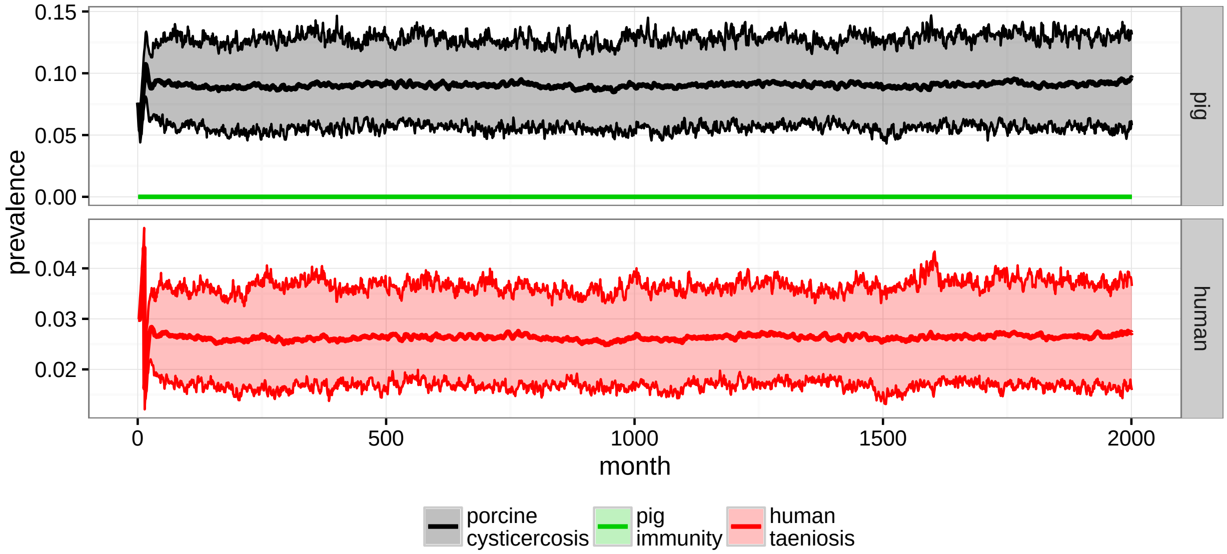

Figure 2: Mbozi baseline model; mean prevalence and 95% uncertainty interval based on 100 simulations of 2000 months each. Parameter settings: $ph2m = 0.00029$; $pl2m = 0.00044$; $m2p = 0.00003$; $e2p = 0.00007$; $age.coef = (-4, 0.02)$.
