# Supplementary material for: CystiSim – An Agent-Based Model for Taenia solium Transmission and Control
Source: PLoS Negl Trop Dis. 2016 Dec 16;10(12):e0005184. doi: 10.1371/journal.pntd.0005184 (PMC5161321; doi:10.1371/journal.pntd.0005184)
Supplement: S2 Appendix — (PDF) [file pntd.0005184.s003.pdf]

cystiSim—an agent-based model for *Taenia solium* transmission and control

S2 Appendix. Mbozi intervention and elimination models.

**ID**	**Strategy**	**Human age group**	**Coverage**	**Porcine cysticercosis** $Pr(elimination)$	**Human taeniosis** $Pr(elimination)$
INT-1	human MDA 4q12	All	75%	0.000	0.000
INT-2	human MDA 4q12	SAC	90%	0.000	0.000
INT-3	pig MDA 11q4	All	90%	0.868	0.864
INT-4	pig MDA 11q4	All	75%	0.541	0.536
INT-5	pig MDA&VAC 11q4	All	90%	0.979	0.979
INT-6	pig MDA&VAC 11q4	All	75%	0.771	0.767
INT-7	human MDA 4q12 + pig MDA 11q4	SAC	90% (Humans)<br>75% (Pigs)	0.665	0.653
INT-8	human MDA 4q12 + pig MDA 11q4	All	75%	0.862	0.856
INT-9	human MDA 4q12 + pig MDA&VAC 11q4	SAC	90% (Humans)<br>75% (Pigs)	0.844	0.835
INT-10	human MDA 4q12 + pig MDA&VAC 11q4	All	75%	0.947	0.943

Table 1: Probability of *Taenia solium* elimination in human and porcine hosts in Mbozi district by simulation in **cystiSim**. Efficacy of was fixed at 90%. MDA: Mass Drug Administration; VAC: Vaccination; SAC: School-Age Children; xqy: x treatments with y months interval.

**ID**	**Strategy**	**Human age group**	**Porcine cysticercosis, months till elimination** *mean (range)*	**Human taeniosis, months till elimination** *mean (range)*
ELIM-1	Human MDA	All	313 (119–1080)	310 (120–1080)
ELIM-2	Pig MDA	Not applicable	49 (24–120)	51 (27–124)
ELIM-3	Pig MDA+VAC	Not applicable	39 (20–88)	43 (24–90)
ELIM-4	Human MDA & Pig MDA+VAC	SAC	38 (20–94)	41 (23–107)
ELIM-5	Human MDA & Pig MDA+VAC	All	30 (14–84)	33 (17–79)

Table 2: Duration of months until elimination of *Taenia solium* in human and porcine hosts was achieved in Mbozi district by simulation in **cystiSim**. Efficacy of was fixed at 90%. Coverage of mass drug administration (MDA) to school-age children (SAC) was set to 90% and for MDA to the whole human population (All) and for pig interventions to 75%. MDA: Mass Drug Administration; VAC: Vaccination; SAC: School-Age Children; xqy: x treatments with y months interval.

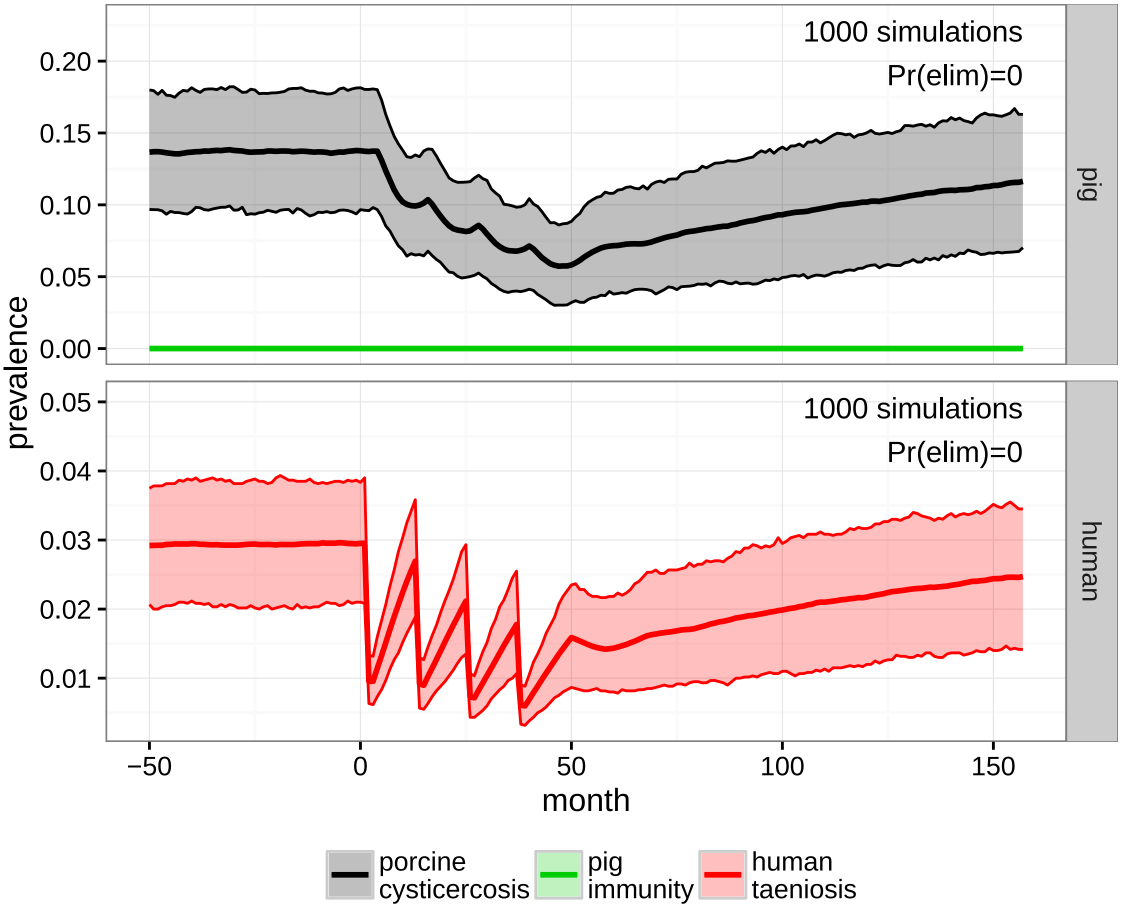

Figure 1: Outcome of the MDA to the whole population with 75% coverage for Mbozi district after 1000 simulations in **cystiSim**. Efficacy was set at 90%. The coloured area demarcates the 95% uncertainty intervals for prevalence. The green line illustrates pig immunity towards new infections and Pr(elim) states the predicted probability of elimination occurring in the given scenario.

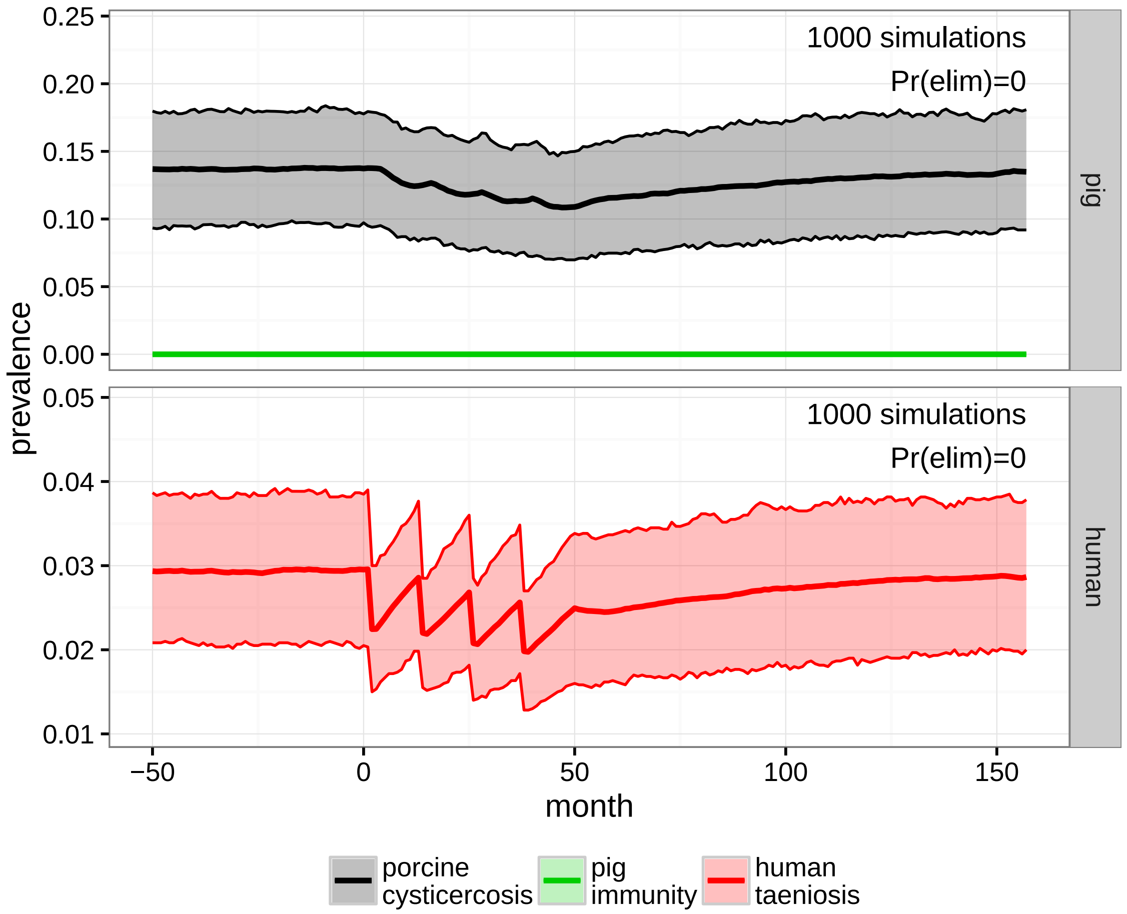

Figure 2: Outcome of the MDA to school-aged children with 90% coverage for Mbozi district after 1000 simulations in **cystiSim**. Efficacy was set at 90%. The coloured area demarcates the 95% uncertainty intervals for prevalence. The green line illustrates pig immunity towards new infections and Pr(elim) states the predicted probability of elimination occurring in the given scenario.

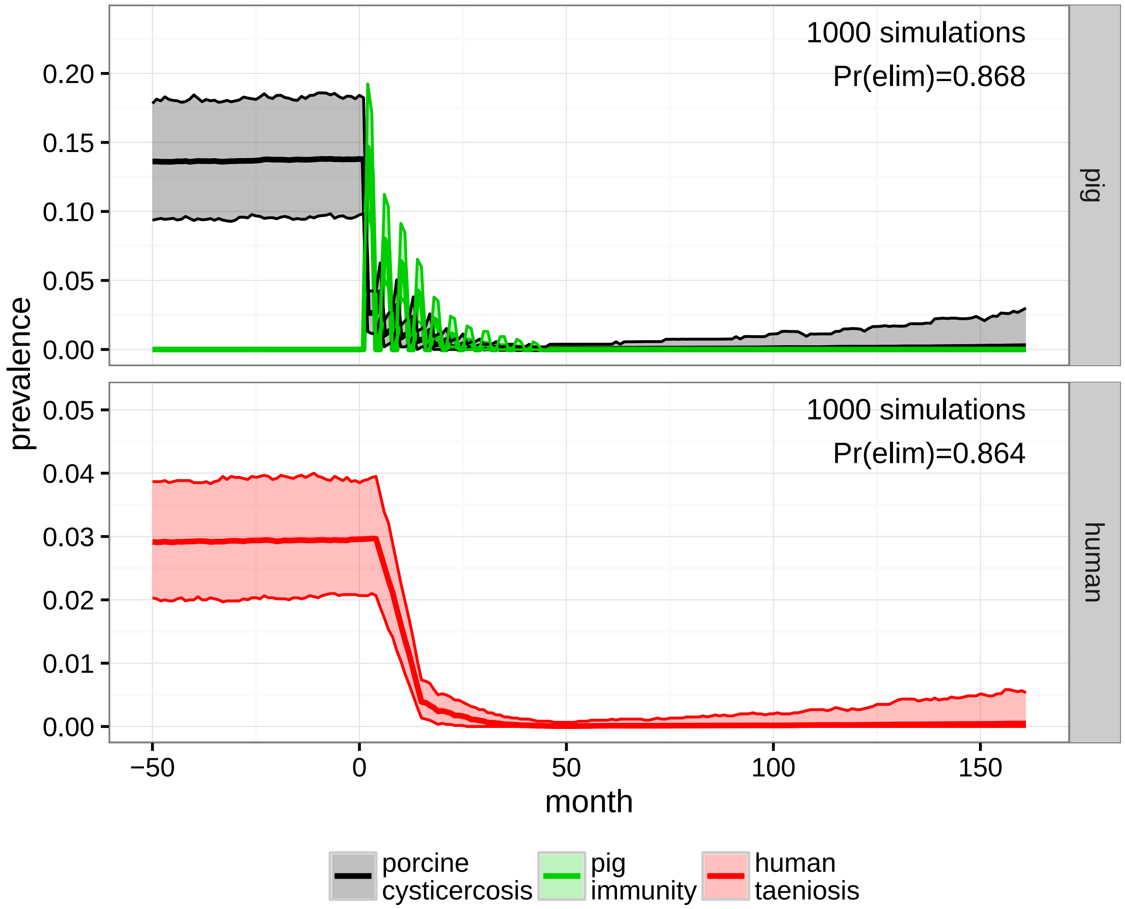

Figure 3: Outcome of the porcine population treatment with 90% coverage for Mbozi district after 1000 simulations in **cystiSim**. Efficacy was set at 90%. The coloured area demarcates the 95% uncertainty intervals for prevalence. The green line illustrates pig immunity towards new infections and Pr(elim) states the predicted probability of elimination occurring in the given scenario.

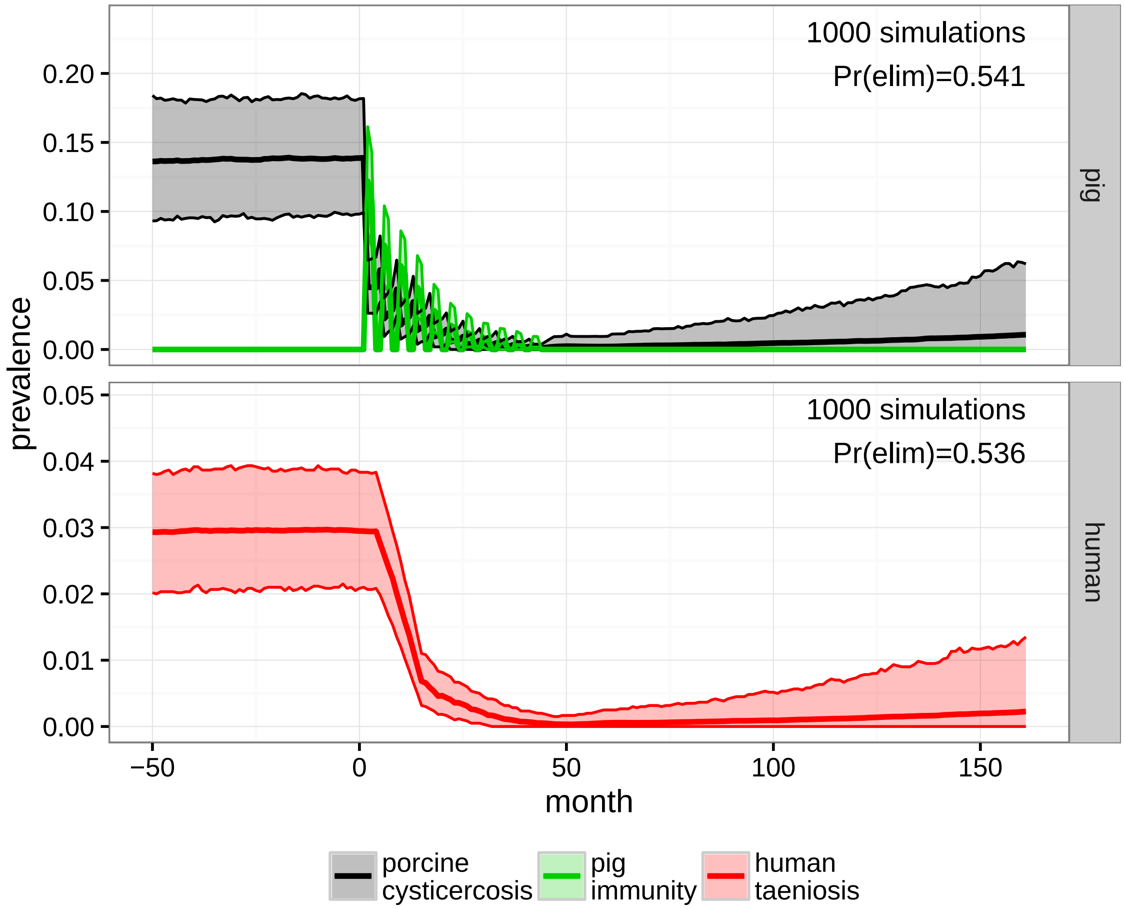

Figure 4: Outcome of the porcine population treatment with 75% coverage for Mbozi district after 1000 simulations in **cystiSim**. Efficacy was set at 90%. The coloured area demarcates the 95% uncertainty intervals for prevalence. The green line illustrates pig immunity towards new infections and Pr(elim) states the predicted probability of elimination occurring in the given scenario.

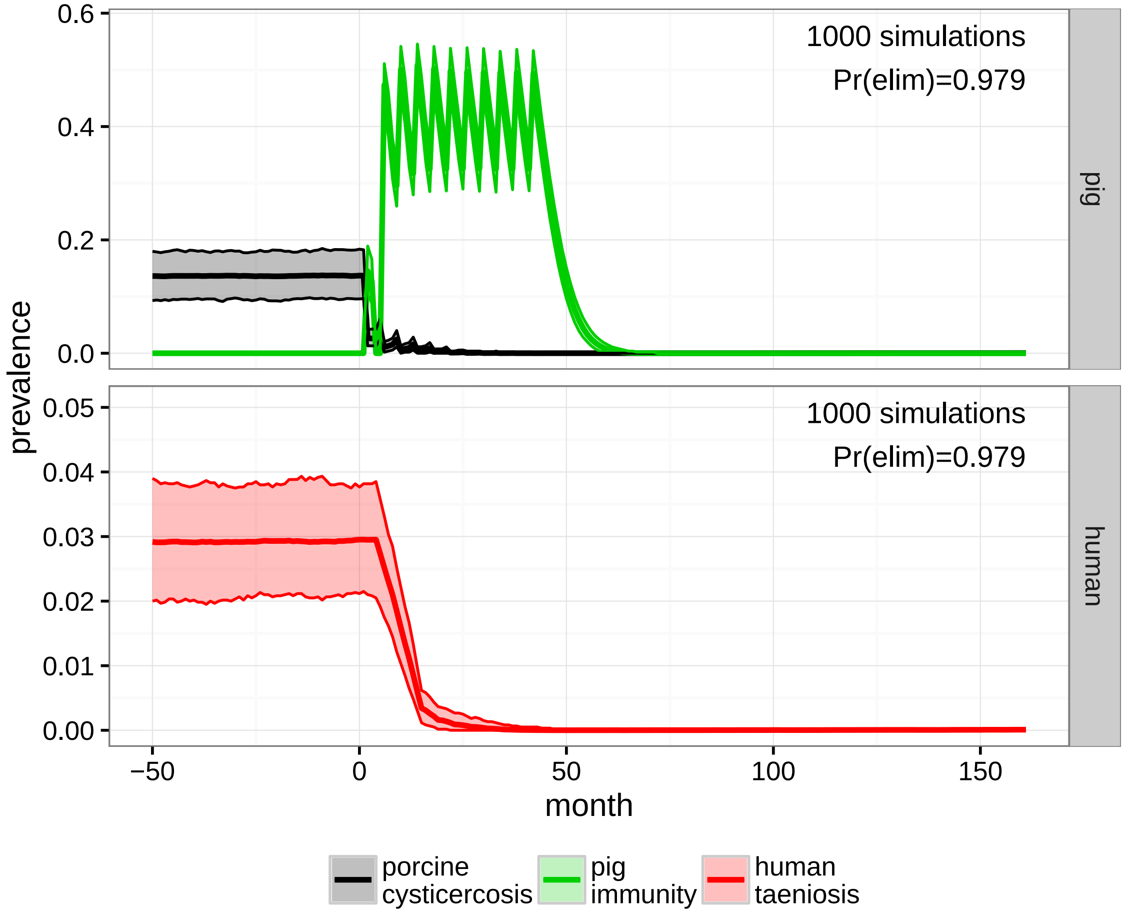

Figure 5: Outcome of anthelmintic treatment and vaccination of the porcine population with 90% coverage for Mbozi district after 1000 simulations in **cystiSim**. Efficacy was set at 90%. The coloured area demarcates the 95% uncertainty intervals for prevalence. The green line illustrates pig immunity towards new infections and Pr(elim) states the predicted probability of elimination occurring in the given scenario.

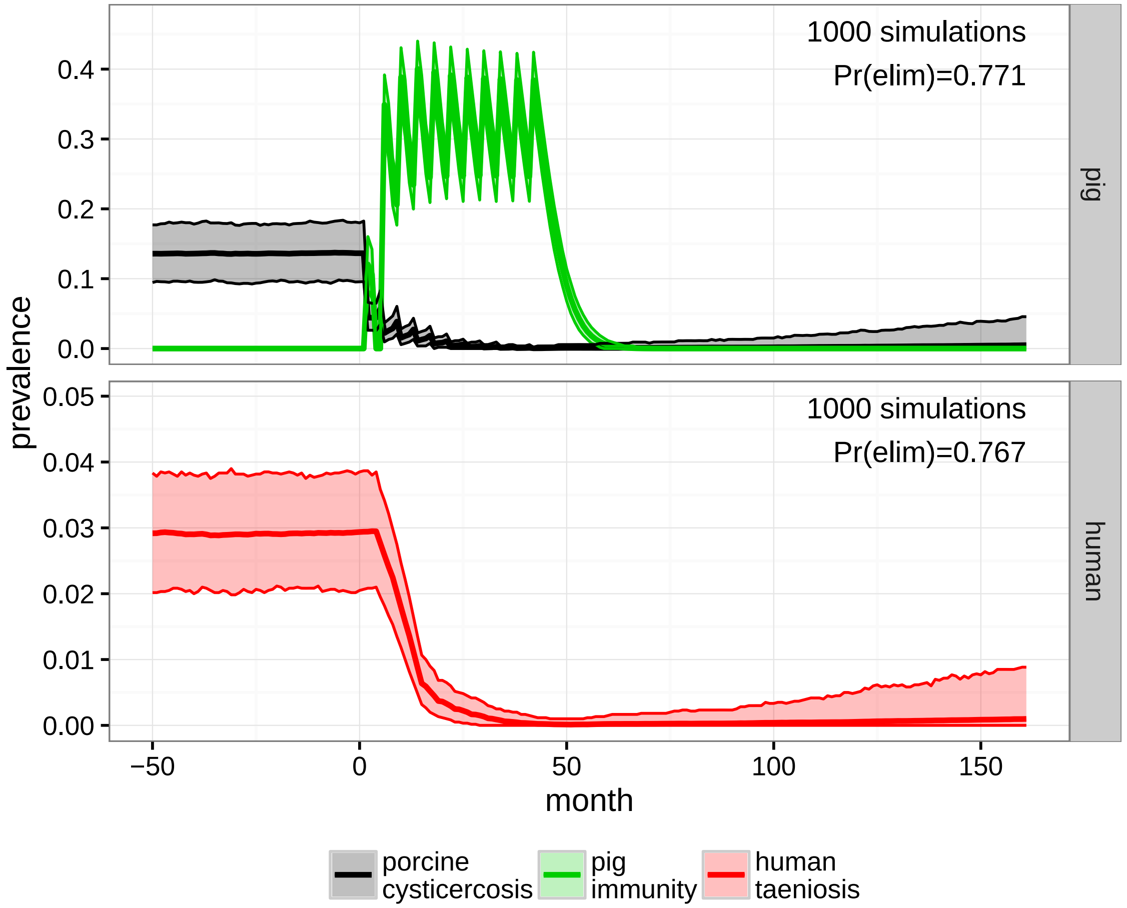

Figure 6: Outcome of anthelmintic treatment and vaccination of the porcine population with 75% coverage for Mbozi district after 1000 simulations in **cystiSim**. Efficacy was set at 90%. The coloured area demarcates the 95% uncertainty intervals for prevalence. The green line illustrates pig immunity towards new infections and Pr(elim) states the predicted probability of elimination occurring in the given scenario.

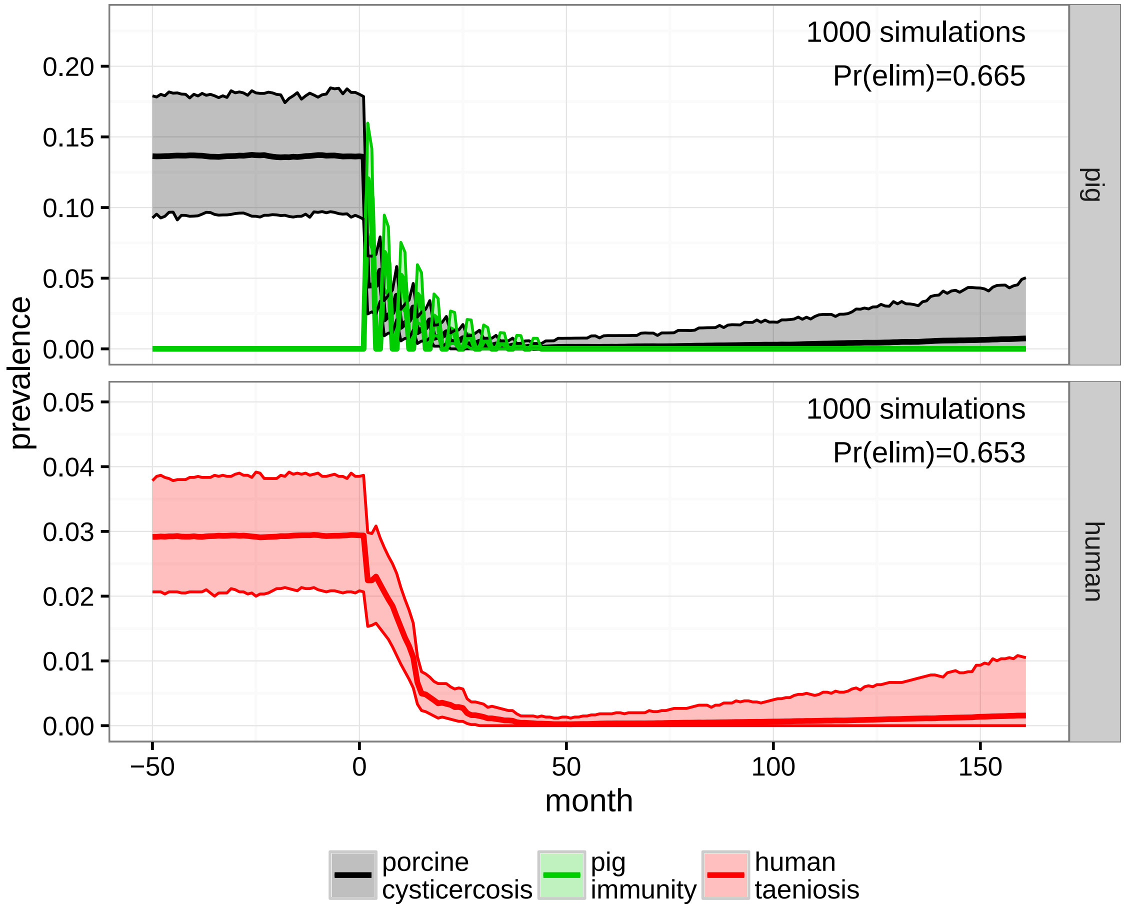

Figure 7: Outcome of MDA to school-aged children with 90% coverage in combination with anthelmintic treatment of the porcine population with 75% coverage for Mbozi district after 1000 simulations in **cystiSim**. Efficacy was set at 90%. The coloured area demarcates the 95% uncertainty intervals for prevalence. The green line illustrates pig immunity towards new infections and Pr(elim) states the predicted probability of elimination occurring in the given scenario.

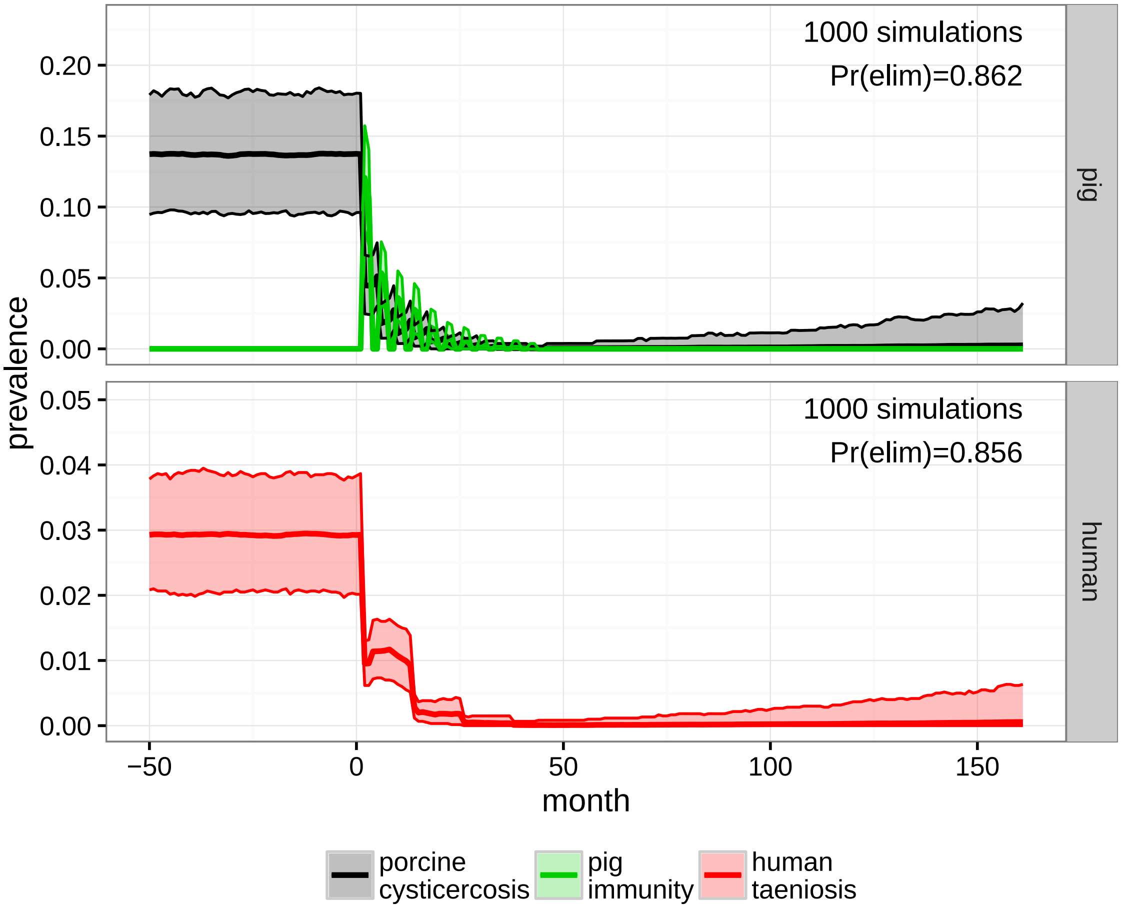

Figure 8: Outcome of MDA to the whole human population in combination with anthelmintic treatment of the porcine population both with 75% coverage (on the right) for Mbozi district after 1000 simulations in **cystiSim**. Efficacy was set at 90%. The coloured area demarcates the 95% uncertainty intervals for prevalence. The green line illustrates pig immunity towards new infections and Pr(elim) states the predicted probability of elimination occurring in the given scenario.

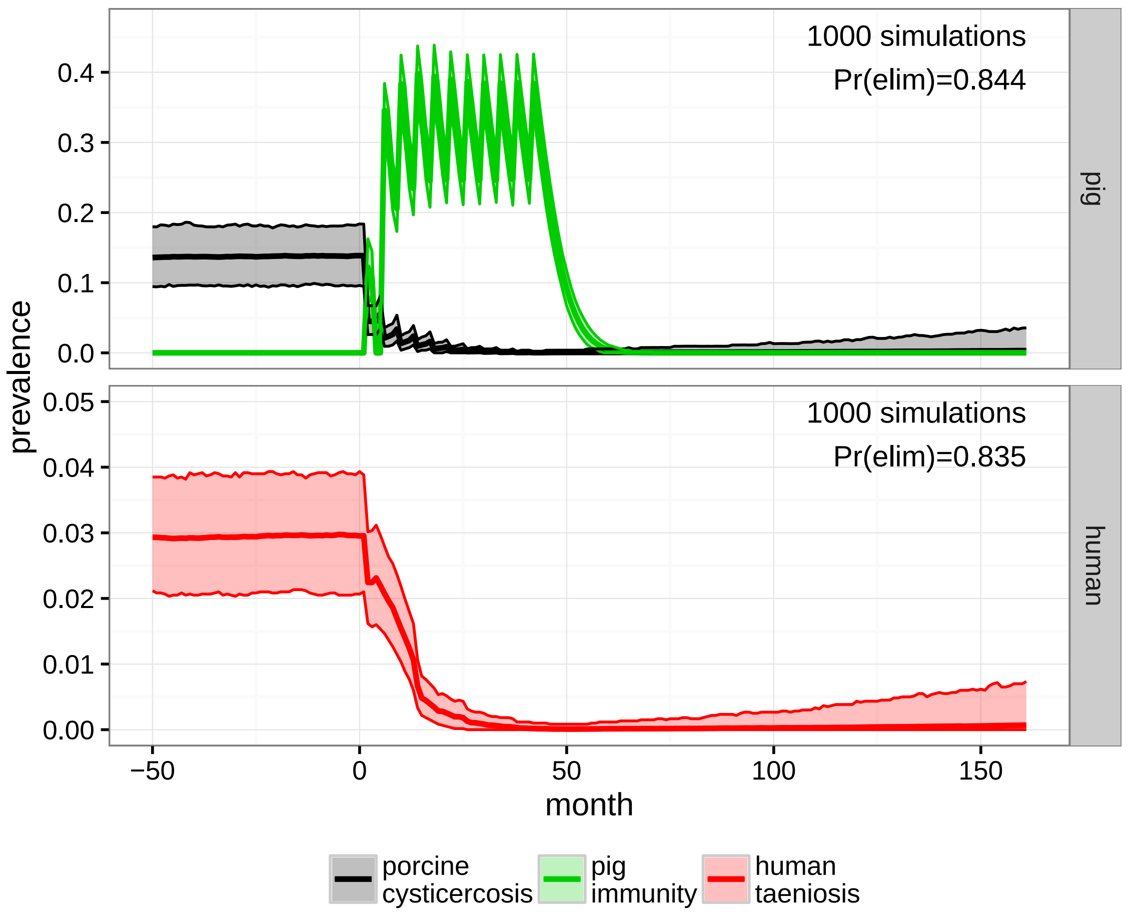

Figure 9: Outcome of MDA to school-aged children with 90% coverage in combination with anthelmintic treatment and vaccination of the porcine population with 75% coverage for Mbozi district after 1000 simulations in **cystiSim**. Efficacy was set at 90%. The coloured area demarcates the 95% uncertainty intervals for prevalence. The green line illustrates pig immunity towards new infections and Pr(elim) states the predicted probability of elimination occurring in the given scenario.

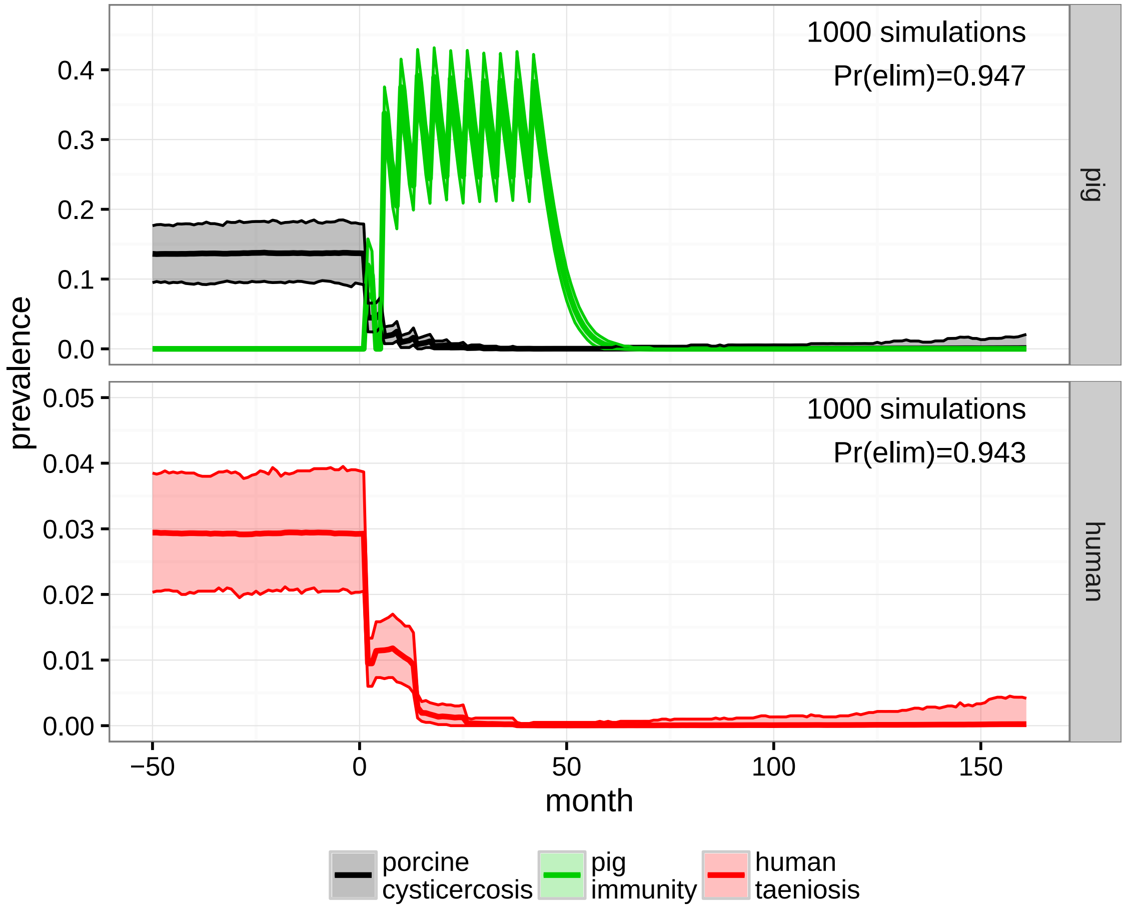

Figure 10: Outcome of MDA to the whole human population in combination with anthelmintic treatment and vaccination of the porcine population both with 75% coverage for Mbozi district after 1000 simulations in **cystiSim**. Efficacy was set at 90%. The coloured area demarcates the 95% uncertainty intervals for prevalence. The green line illustrates pig immunity towards new infections and Pr(elim) states the predicted probability of elimination occurring in the given scenario.
